# Supplementary material for: Comparison of Quantification Methods to Estimate Farm-Level Usage of Antimicrobials Other than in Medicated Feed in Dairy Farms from Québec, Canada
Source: Microorganisms. 2021 May 20;9(5):1106. doi: 10.3390/microorganisms9051106 (PMC8160742; doi:10.3390/microorganisms9051106)
Supplement: Supplementary file 1 [file microorganisms-09-01106-s001.zip › microorganisms-1190982 - Table S2 FINAL.pdf]

Table S2. Demographic data (number of farms recruited by region, herd size, and owned quota) of the 101 dairy farms recruited in the Québec province of Canada.

Demographic data collected	Montérégie	Centre-du-Québec	Estrie	Total
Number of farms recruited	45	34	22	101
Herd size in number of adult dairy cows[1] per farm (median (min, max))	65 (20, 150)	59 (30, 98)	62 (35, 106)	61 (20, 150)
Herd size in total number of cattle per farm (median (min, max))	109 (39, 260)	105 (50, 192)	103 (51, 175)	107 (39, 260)
Owned quota in kg of fat content per day (median (min, max))	73 (20, 174)	58 (19, 101)	59 (34, 110)	65 (19, 174)

[1] Including lactating cows and dry cows.

References

1. Lardé, H.; Dufour, S.; Archambault, M.; Massé, J.; Roy, J.-P.; Francoz, D. An observational cohort study on antimicrobial usage on dairy farms in Quebec, Canada. *J. Dairy Sci.* **2021**, *104*, 1864-1880, doi:10.3168/jds.2020-18848.
