# Supplementary material for: Comparison of Quantification Methods to Estimate Farm-Level Usage of Antimicrobials Other than in Medicated Feed in Dairy Farms from Québec, Canada
Source: Microorganisms. 2021 May 20;9(5):1106. doi: 10.3390/microorganisms9051106 (PMC8160742; doi:10.3390/microorganisms9051106)
Supplement: Supplementary file 1 [file microorganisms-09-01106-s001.zip › microorganisms-1190982 - Table S3 FINAL.pdf]

Table S3. Antimicrobial agents identified by at least one quantification method (REF, VET, GOV, or FARM) in 101 (REF, VET, and GOV methods) or 97 (FARM method) dairy farms from Québec, Canada, but not quantified in Canadian Defined Course Doses for cattle (DCDbovCA). Products containing antimicrobial agents that could not be reported in DCDbovCA were rarely identified by the four methods of quantification, but should be mentioned here, as they were not taken into account for the analyses. Three types of products were concerned (with no equivalent labeled for cattle): compounded forms (CF), veterinary products labeled for species different from cattle or no more labeled for cattle (VP), and human products (HP). Note that several CF containing oxytetracycline for intrauterine infusion were identified but could be converted in number of DCDbovCA as the DCDbovCA value for oxytetracycline used through the uterine route was assigned (1 DCDbovCA = 2.5 grams per animal and per course) [26]. Similarly, ampicillin sodium from HP (powder for injection) could be reported in number of DCDbovCA as the DCDbovCA value for ampicillin used through the injectable route was assigned (1 DCDbovCA = 19.5 grams per animal and per course) [26]. Globally, a higher usage of these specific products was identified using quantification from the VET method in comparison with REF, GOV, and FARM methods (Table S3). It was hypothesized that products labeled for species other than cattle and present in veterinary invoices could be bought and used for the non-bovine animals of the farm. Also, for ophthalmic formulations, no product is currently available in Canada for cattle, forcing veterinarians and producers to use off-label products.

Route of administration	Type of product	Formulation and antimicrobial agents included in the product	REF method (101 farms)	VET method (101 farms)	GOV method (101 farms)	FARM method (97 farms – excluded farms: #14-46-70-87)
Intramammary	CF	Solution containing Lincomycin 6 mg/mL	1 farm #62	1 farm #62	1 farm #62	1 farm #62
Intramammary	CF	Solution containing Oxytetracycline 12 mg/mL	1 farm #85	5 farms #62-74-75-85-95	4 farms #74-75-85-95	2 farms #62-85
Injectable	CF	Solution containing Spectinomycin 100 mg/mL	1 farm #04	1 farm #79	1 farm #79	1 farm #79
Injectable	CF	Solution containing Spectinomycin 88.9 mg/mL and Lincomycin 44.4 mg/mL	1 farm #48	1 farm #48	-	-
Injectable	VP	Solution containing Spectinomycin 100 mg/mL and Lincomycin 50 mg/mL (**Linco-Spectin® Sterile Solution**), labeled for cats and dogs	1 farm #60	3 farms #09-60-69	2 farms #09-69	3 farms #31-75-85
Injectable	VP	Solution containing Dihydrostreptomycin 500 mg/mL (**Ethamycin® Liq 500**), cancelled post-market (previously labeled for cattle)	-	-	-	2 farms #05-65
Injectable	HP	Powder for injection containing Penicillin G sodium 1 million/vial	1 farm #33	3 farms #08-17-33	3 farms #08-17-33	-
Injectable	HP	Powder for injection containing Penicillin G sodium 5 millions/vial	2 farms #08-20	8 farms #06-07-08-14-20-21-28-50	8 farms #06-07-08-14-20-21-28-50	1 farm #07
Injectable	HP	Powder for injection containing Penicillin G sodium 10 millions/vial	4 farms #01-14-26-88	8 farms #01-04-15-25-26-27-41-88	7 farms #01-04-15-25-26-41-88	-
Injectable	HP	Powder for injection containing Erythromycin Lactobionate 1g/vial	-	1 farm #22	-	-
Injectable	HP	Powder for injection containing Cefazolin sodium 1g/vial	-	1 farm #97	-	-

Oral	CF	Water soluble powder containing Enrofloxacin 40 mg/g	-	1 farm #48	-	-
Oral	CF	Water soluble powder containing Doxycycline 48 mg/g and Oxolinate 48 mg/g	-	1 farm #48	-	-
Oral	CF	Water soluble powder containing Sulfadiazine 82.3 mg/g and Tylosin Tartrate 100 mg/g	-	1 farm #48	-	-
Oral	VP	Water soluble powder containing Spectinomycin 444 mg/g and Lincomycin 222 mg/g (**Linco-Spectin® 100 Soluble Powder**), labeled for swine and poultry	4 farms #31-75-78-85	3 farms #75-78-85	3 farms #75-78-85	-
Oral	VP	Water soluble powder containing Lincomycin 400 mg/g (**Lincomix®**), labeled for chickens, swine, and honey bees	1 farm #57	2 farms #52-79	1 farm #79	-
Oral	VP	Solution containing Spectinomycin 50 mg/mL (**Spectam® Oral Solution**), labeled for swine	-	2 farms #70-77	2 farms #70-77	-
Oral	VP	Suspension containing Chloramphenicol 50 mg/mL (**ChlorPalm 250®**), labeled for cats and dogs	-	1 farm #91	# farm #91	-
Oral	VP	Tablets containing Amoxicillin 40 (or 50) mg/tablet and Clavulanic acid 10 (or 12.5) mg/tablet (**Clavaseptin®**), labeled for cats and dogs	-	1 farm #80	1 farm #80	-
Oral	HP	Tablets containing Trimethoprim 160 mg/tablet and Sulfamethoxazole 800 mg/tablet	1 farm #41	4 farms #01-13-19-41	4 farms #01-13-19-41	-
Oral	HP	Tablets containing Amoxicillin 500 mg/tablet	-	1 farm #65	1 farm #65	-
Topical	CF	Topical ointment containing Tetracycline 200 mg/g	-	1 farm #15	-	1 farm #42
Topical	VP	Topical ointment containing Nitrofurazone 2 mg/g (**Nitro Ointment®**), labeled for horses	-	1 farm #11	-	1 farm #01
Topical	VP	Topical and otic suspension containing Polymyxin B Sulfate 0.5293 mg/mL (and Miconazole Nitrate, and Prednisolone acetate) (**Surolan®**), labeled for cats and dogs	-	1 farm #63	-	-
Topical	VP	Topical and otic solution containing Neomycin 3.2 mg/mL (and Thiabendazole, and Dexamethazone) (**Tresaderm®**), labeled for cats and dogs	-	1 farm #87	1 farm #87	-
Topical	HP	Ophthalmic solution containing Tobramycin 3 mg/mL (**Tobrex®**)	-	2 farms #43-96	2 farms #43-96	-
Topical	HP	Ophthalmic ointment containing Neomycin 3.5 mg/g, polymyxin B sulfate 6,000 IU/g (and dexamethasone) (**Maxitrol®**)	-	1 farm #05	1 farm #05	-

References

1. Lardé, H.; Dufour, S.; Archambault, M.; Léger, D.; Loest, D.; Roy, J.-P.; Francoz, D. Assignment of Canadian Defined Daily Doses and Canadian Defined Course Doses for Quantification of Antimicrobial Usage in Cattle. Front. Vet. Sci. **2020**, *7*, 10, doi:10.3389/fvets.2020.00010.
